# Auditory selective attention in depth: Investigating directional dependency across front, lateral, and rear spaces

**DOI:** 10.3758/s13414-026-03292-x

**Published:** 2026-06-23

**Authors:** Ryo Teraoka, Yuki Tanaka, Wataru Teramoto

**Affiliations:** 1 Graduate School of Engineering, Muroran Institute of Technology, 27-1 Mizumoto-cho, Muroran, Hokkaido 050-8585 Japan; 2 Graduate School of Social and Cultural Sciences, Kumamoto University, 2-40-1 Kurokami, Kumamoto, 860-8555 Japan; 3 Faculty of Humanities and Social Sciences (Psychology), Kumamoto University, 2-40-1 Kurokami, Kumamoto, 860-8555 Japan

**Keywords:** Auditory distance perception, Auditory selective attention, Spatial attention, Peripersonal space, Cocktail-party effect

## Abstract

Auditory spatial attention enables listeners in complex acoustic environments to focus on relevant sounds while filtering out irrelevant noise. However, its mechanisms in the depth dimension remain unclear, especially across different horizontal directions. This study examined whether the effect of auditory selective attention in the depth dimension varies across the front, lateral, and rear spaces under reverberant listening conditions. In the experiment, listeners detected target sounds at one of five distances (32, 64, 96, 128, and 160 cm) under conditions where attention was either distributed or focused on a specific distance. The results showed that sensitivity (*d′*) was significantly enhanced at the attended distance across all directional conditions, with no statistically significant differences in the magnitude of attentional enhancement between the front, lateral, and rear directions. Conversely, the reaction time (RT) revealed limited attentional benefits, emerging only at specific distances in the lateral and rear spaces. These findings demonstrated that auditory selective attention in the depth dimension facilitates target detection irrespective of horizontal directions, supporting the generalizability of attentional mechanisms across the depth dimension of auditory space, at least under reverberant listening conditions.

**Supplementary Information:**

The online version contains supplementary material available at 10.3758/s13414-026-03292-x.

## Introduction

In everyday life, listeners are frequently exposed to complex acoustic environments with multiple competing sounds—for instance, when conversing with a friend in a bustling café or reacting to auditory cues while crossing a busy street. Even in such situations, humans have the remarkable ability of focusing on a particular sound while suppressing irrelevant background noise, referred to as the “cocktail-party effect” (Cherry, 1953). Previous studies have shown that listeners utilize a variety of acoustic features, including spatial location, fundamental frequency, temporal cues, and dynamic voice characteristics, to segregate and attend to a sound of interest (Bronkhorst, 2000, 2015; Ebata, 2003). Among these cues, spatial information is particularly important for segregating and selecting target sounds within complex acoustic environments, especially in cocktail-party situations, wherein multiple sound sources are often present simultaneously (Bronkhorst, 2000; Pastore & Yost, 2017; Yost, 2017).

Although most previous studies on auditory spatial attention have focused on azimuthal directions, attention must be allocated in both azimuth and depth in everyday listening situations. For example, hearing an approaching vehicle from behind or orienting to a voice coming from a distant location. Despite its ecological importance, auditory attention in the depth dimension remains largely unexplored. Previous studies from our research group have demonstrated that directing auditory attention to a specific distance could enhance perceptual sensitivity for the target sound, particularly in complex listening situations (Monasterolo, 2019; Sakamoto et al., 2019; Teraoka et al., 2025). Teraoka et al. (2025) investigated whether auditory attention directed to a specific distance modulates listening performance in complex auditory scenes using sounds presented from the front space; they found that target detection was enhanced and responses to distractors were reduced at the attended distance, suggesting that auditory selective attention operates in the depth dimension.

Most studies examining auditory spatial attention, whether in the horizontal or depth dimension, have primarily focused on the front space. However, one of the unique advantages of the human auditory system is its ability to detect and process sounds from all directions around the body, including regions outside the visual field. As direct visual monitoring is limited or unavailable in these regions, rapid and accurate auditory processing beyond the front space is essential for safety and effective interaction. Few studies have directly examined whether the spatial role of auditory selective attention varies across directions beyond the front space (Bodnár et al., 2025; Teder-Sälejärvi et al., 1999). Teder-Sälejärvi et al. (1999) compared the spatial profiles of auditory attention between the front and lateral spaces and found a shallower attentional gradient in the lateral direction, suggesting that attention is more diffusely distributed in that direction. Bodnár et al. (2025) examined whether the spatial profile of auditory selective attention differs between the front and rear spaces and reported a steeper attentional gradient in the front than in the rear space. Collectively, these findings suggest that the spatial profile of auditory selective attention varies across directions on the horizontal plane. However, whether such directional differences extend to selective attention in the depth dimension across the front, lateral, and rear spaces remains unclear.

Furthermore, recent studies have indicated that sounds presented behind the listener may be processed differently from those presented in front (e.g., Asutay & Västfjäll, 2015; Olszanowski et al., 2023). Asutay and Västfjäll (2015) examined how sounds presented outside the visual field influence attentional, emotional, and behavioral responses; they reported faster and more accurate localization for sounds presented behind the listener, along with stronger negative emotional evaluations, suggesting an attentional bias toward the rear space. Although these findings do not directly address the spatial profile of auditory selective attention, they imply that auditory processing in the rear space may differ from that in the front space, suggesting that the rear space may be functionally distinct.

The present study aimed to investigate whether auditory attention in the depth dimension differs across the front, lateral, and rear spaces, using sensitivity (*d′*) and reaction time as indices. Previous studies have demonstrated that auditory spatial attention in the azimuthal dimension is distributed in a spatially graded manner, with attentional effects peaking at the attended location and decreasing with increasing distance from that location. If attention in depth is governed by a similar spatially selective mechanism across directions, the spatial profile of attention should be comparable across the front, lateral, and rear spaces, with attentional effects peaking at the attended distance and declining with increasing distance from it. Conversely, prior findings have indicated that auditory spatial attention on the horizontal plane may vary as a function of direction, such that the magnitude and spatial profile of attentional modulation may not be identical across the front, lateral, and rear spaces. These findings further suggest that directional differences in auditory spatial processing—such as the spatial resolution and precision with which sound locations are represented—may influence the attentional gradient’s characteristics. If such direction-dependent characteristics extend to auditory attention in depth, both the magnitude and spatial extent of attentional modulation may differ across directions. To examine this issue, we employed a probability-based procedure^1^ wherein one distance was presented more frequently than the others and compared the resulting modulation of performance across the front, lateral, and rear spaces.

Furthermore, to evaluate the effect of attention in depth, it is crucial to confirm whether there are differences in the auditory distance perception between tested spaces. Previous studies have revealed that auditory spatial representations are not isotropic around the body, with variations in spatial resolution across the frontal, lateral, and rear spaces (Aggius-Vella et al., 2020). Moreover, auditory distance perception has been demonstrated to be less precise for sounds originating from the rear space than from the front space (Aggius-Vella et al., 2022). Therefore, the present study measures the auditory distance perception at the same positions and spaces as the main task, to distinguish attentional effects from possible perceptual biases in distance perception.

## Methods

### Listeners

A total of 16 undergraduate and graduate students (six men; mean age = 21.82 ± 2.04 [*SD*] years) were included. The sample size was determined by a priori power analysis using G*Power 3.1.9.4 (Faul et al., 2009), which indicated that a sample size of 11 listeners was required (α error probability = 0.05, power [1 − β error probability] = 0.95; repeated-measures within-subjects design) for a medium effect size (*f* = 0.25; Cohen, 1992). In a previous study (Teraoka et al., 2025) employing a similar experimental design, approximately 22% of listeners were excluded owing to incomplete participation or failure to provide valid responses in certain conditions. Considering this exclusion rate, we recruited 16 listeners to ensure sufficient data for analysis. All listeners had normal hearing and were naïve to the purpose of the experiment. This study was approved by the Ethics Committee of the Faculty of Humanities and Social Sciences, Kumamoto University and was conducted in accordance with the principles of the Declaration of Helsinki (1964) and its updated version (2013). The listeners provided written informed consent before commencing the experiment.

### Apparatus and stimuli

**Fig. 1:**
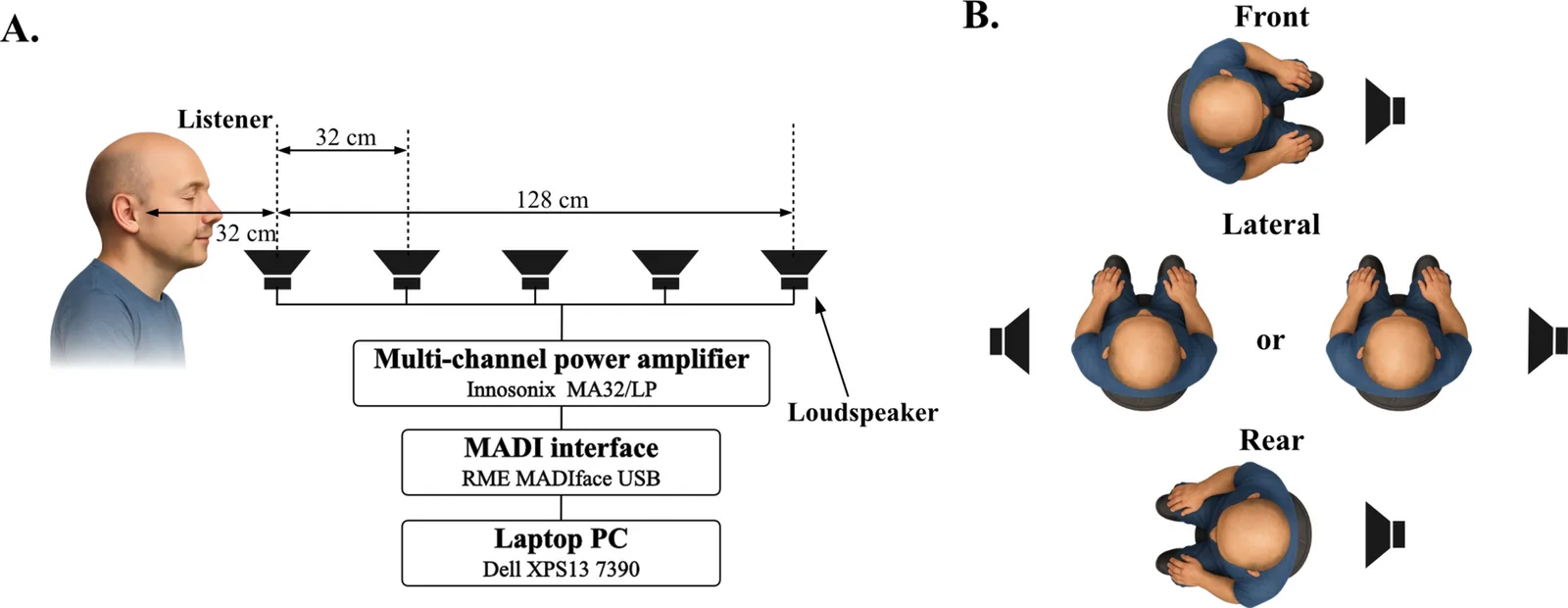
Schematic illustration of the experimental setup. **A**. Five loudspeakers were placed at intervals of 32 cm, ranging from 32 cm to 160 cm, along the depth dimension from the listener’s head. **B.** Tested directions were front (0°), lateral (90° or 270°), and rear (180°). The listener’s orientation varied across conditions such that the sound stimuli were presented from the front, lateral, and rear

The experimental apparatus, room layout, and stimuli were identical to those used by Teraoka et al. (2025). A schematic of the experimental setup is shown in Fig. 1A. The sound stimuli were delivered via five loudspeakers (Aura Sound, NSW1-205-A) arranged along the depth dimension in a quiet room. The loudspeakers were installed at chest level (110 cm in height) and faced upward toward the ceiling to minimize sound occlusion by the loudspeaker in front. The nearest loudspeaker was positioned 32 cm from the center of the listener’s head, and the remaining four loudspeakers were placed at 32-cm intervals, positioned at distances of 64, 96, 128, and 160 cm from the listener.

The experiment was conducted in a rectangular room measuring 5.96 m (width) × 10.82 m (length) × 2.99 m (height). The room had painted walls and woven tile carpeting. Owing to spatial constraints, the loudspeaker array was located approximately 1.75 m from the nearest side wall and 1.25 m from the wall behind the array. The room was not acoustically treated (i.e., not anechoic), and acoustic reflections from the floor, ceiling, walls, and experimental equipment were present. However, the spatial layout and acoustic environment were kept constant across all trials and experimental conditions.

The sound stimuli were controlled using a laptop computer (Dell XPS 7390) with a MADI interface (RME MADIface USB) and a multi-channel power amplifier (Innosonix MA32/LP) using GNU Octave (Version 6.2.0) with Playrec (Version 6.2.0) and Psychtoolbox 3.0 (Brainard, 1997; Kleiner et al., 2007; Pelli, 1997). A numeric keypad was used to record the responses.

The sound stimulus was an amplitude-modulated pink noise burst (duration: 200 ms, including 5-ms rise and fall times), which was used in previous studies (Golob & Mock, 2020; Teraoka et al., 2025). The sounds were amplitude-modulated at either 25 Hz or 75 Hz (90% modulation depth), with 25-Hz-modulated sounds serving as targets and 75-Hz-modulated sounds as distractors. This modulation provided a nonspatial cue for distinguishing the sound type, while all stimuli contained the full spectral range required for localization and were equalized in energy across the sounds. The equivalent continuous A-weighted sound pressure level (*L*_Aeq_) was set to 70 dB at 32 cm in front of the listener, measured at the center of the listener’s head in their absence. The same electrical input level was applied to all loudspeakers, thereby decreasing the sound pressure level at the listener’s head with increasing loudspeaker distance.

### Procedure

The experiment comprised one main and one supplementary task: auditory attention and distance perception. Each task was performed in three spaces: 0° (front), 90° or 270° (lateral), and 180° (rear). For the lateral condition, either the left (90°) or right (270°) direction was assigned to each listener (90° for half of the listeners and 270° for the other half); this assignment was counterbalanced across listeners to reduce fatigue. The direction conditions were implemented by physically orienting the listener’s body relative to the loudspeaker array. Each space was tested in a different session. The listeners were asked to keep their head stationary and close their eyes when sound stimuli were presented.

#### Auditory attention task

This task included the following two conditions: no-focus and focus. In both conditions, a sound stimulus was presented from one of the five loudspeakers on each trial. The inter-stimulus interval (ISI) was jittered within a range of 500 to 1000 ms (mean: 750 ms) to prevent temporal predictability. The distance of the active loudspeaker was randomly selected on each trial, with the constraint that the same distance was not presented more than twice consecutively. Target sounds were presented on 20% of trials and distractor sounds on 80%. Listeners were instructed to respond as quickly as possible upon detecting a target sound and to withhold responses to distractor sounds. To mitigate fatigue, each condition was divided into three equal-length blocks, with short breaks provided between blocks while maintaining a uniform distribution of target and distractor trials.

The task began with a brief practice trial (10 trials) to familiarize listeners with the procedure. During this practice period, the experimenter confirmed that listeners could reliably distinguish between target and distractor sounds.

In the no-focus condition, stimulus presentation probabilities were equal across all five loudspeakers (*p* =.20). This manipulation ensured that no specific distance was prioritized through stimulus statistics and that listeners were not guided to attend to any particular distance. Target sounds were also equally likely to be presented from each loudspeaker (*p* =.20). Each block began with a 500-ms, 1000-Hz pure tone (with 5-ms rise/fall times) presented simultaneously from all the five loudspeakers to indicate block onset. After a 1-s interval, a sound stimulus was presented from one of the loudspeakers. This condition included a total of 225 trials: 45 target trials (5 distances × 9 repetitions) and 180 distractor trials (5 distances × 36 repetitions).

In the focus condition, stimulus presentation probabilities were manipulated across distances using a probability-based procedure adapted from previous auditory attention studies (Arbogast & Kidd, 2000; Teraoka et al., 2021, 2025). This manipulation was designed to bias attentional allocation toward a specific distance through stimulus statistics. In each case, either 32 cm or 160 cm was designated as the focus distance, and the probability of stimulus presentation was adjusted accordingly. Each block began with a 500-ms, 1000-Hz pure tone presented simultaneously from all loudspeakers, followed by a 1-s interval before stimulus onset. Importantly, listeners were not explicitly informed about this probability manipulation and were not instructed to attend to any specific distance. This condition consisted of 900 trials for each focus distance: 144 target trials at the focus distance and 36 target trials distributed across the remaining four distances (4 distances × 9 trials each); 576 distractor trials at the focus distance and 144 distractor trials across the remaining distances (4 distances × 36 trials each).

The orders of conditions (no-focus, and focus), tested directions (front, lateral, and rear), and focus distances within the focus condition (32, and 160 cm) were randomized across listeners.

#### Auditory distance perception task

Each block commenced with a 500-ms pure tone (1000 Hz, 5-ms rise/fall times) presented simultaneously from all five loudspeakers to indicate the start of the block. After a 1-s interval, a 200-ms pink noise burst was presented from one of the five loudspeakers (32, 64, 96, 128, or 160 cm). The pink noise burst was not amplitude-modulated, as this task was designed to assess auditory distance perception independently of the amplitude-modulation cues utilized to define targets and distractors in the main task. The listeners were asked to estimate the distance of the sound stimulus and verbally report it in meters. The task comprised 150 trials with 10 repetitions for each combination of three directions and five distances. The order of loudspeaker presentation was randomized.

#### Data analyses

Statistical analyses were performed using R (Version: 4.4.2) with the *anovakun* package (Iseki, 2020) and JASP (Version 0.19.3). For the auditory attention task, sensitivity (*d′*) based on signal detection theory (Peterson et al., 1954; Tanner & Swets, 1954) and reaction time (RT) were used as indices of attentional effects. Hit and false-alarm (FA) rates were calculated for each listener and condition. A response to a target sound was classified as a hit if it occurred between the onset of the target stimulus and the end of the subsequent ISI; failure to respond within this interval was considered a miss. A response to a distractor sound within the same interval was classified as a FA, whereas no response to a distractor was regarded as a correct rejection.

In cases where the hit or FA rates were 0 or 1 (i.e., perfect or no detection), the log-linear correction was applied to prevent infinite *Z*-score values in the sensitivity computation (Hautus, 1995). Specifically, rates of 0 were replaced with 1/(2*N*), and rates of 1 were replaced with 1 − 1/(2*N*), where *N* equals the number of target or distractor trials. Following this correction, the hit and FA rates were transformed into *z*-scores using the inverse cumulative distribution function of the standard normal distribution, and sensitivity was computed using the following formula: *d′* = *Z*(hit) − *Z*(FA).

In the present setup, sound pressure level decreased with increasing distance. This distance-dependent change in level may have made adjacent sound source distances more discriminable, potentially influencing task performance (e.g., higher accuracy and shorter RTs). To minimize the contribution of these physical effects and isolate attentional effects, we analyzed relative performance by subtracting values in the no-focus condition from those in the focus condition (i.e., Δ*d*′ and ΔRT). Absolute *d′* and RT values for each condition are provided in the Supplementary Materials. Subsequently, we conducted a three-way repeated-measures analysis of variance (ANOVA) on the mean data for each task with attention (2; near, and far), direction (3; front, lateral, and rear), and distance (5; 32, 64, 96, 128, and 160 cm) as within-subject factors. Multiple comparisons were performed using Holm’s correction, a sequentially rejective Bonferroni (SRB) procedure, with the family-wise significance level set at α =.05.

For the auditory distance perception data, a two-way repeated-measures ANOVA was conducted on the mean judged distance (averaged across trials for each listener), with direction (3: front, lateral, and rear) and distance (5: 32, 64, 96, 128, and 160 cm) as within-subject factors. Mauchly’s test was used to assess the assumption of sphericity. When this assumption was violated, degrees of freedom were adjusted using Greenhouse–Geisser’s epsilon. Multiple comparisons were conducted using Holm’s correction with α =.05.

As an exploratory analysis, we examined whether performance in the focus condition deviated from the no-focus baseline for each condition. First, the Shapiro–Wilk test was used to assess the normality of the difference scores (Δ*d*′ and ΔRT). Because normality was violated in some conditions, one-sample Wilcoxon signed-rank tests against zero were conducted for each combination of distance, direction, and attention condition.

## Results

Three listeners were excluded from the analysis because the false alarm rate in at least one distance condition in the no-focus condition exceeded ±3 standard deviations from the across-listener mean for that distance. Consequently, the final analysis was conducted on data from 13 listeners.

To complement the difference scores reported below, absolute *d′* and RT values for each condition are provided in the Supplementary Materials.

### Auditory attention

#### *Sensitivity (*d′*)*

**Fig. 2:**
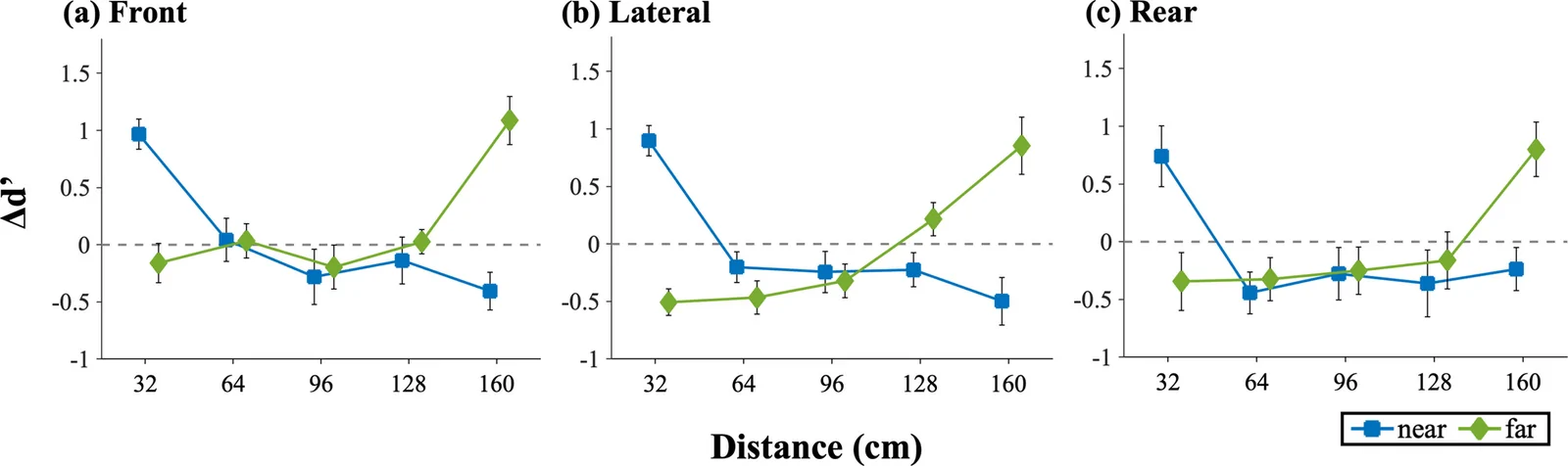
Difference in sensitivity (Δ*d′*) between the focus and no-focus conditions as a function of target distance. **(a)** Front condition, **(b)** lateral condition, and **(c)** rear condition. Error bars represent the standard error of the mean. For better readability, data points are slightly offset along the horizontal axis. (Color figure online)

Figure 2 shows the relative sensitivity (Δ *d′*) as a function of the tested distance for each condition. The ANOVA revealed a main effect of distance, *F*(4, 48) = 8.57, *p* <.001, η_p_^2^ =.417, and interaction between attention and distance, *F*(4, 48) = 96.03, *p* <.001, η_p_^2^ =.889, but no other main effects or interactions, direction: *F*(2, 24) = 1.37, *p* =.274, η_p_^2^ =.102; attention: *F*(1, 12) = 0.87, *p* =.368, η_p_^2^ =.068; direction × attention: *F*(1.35, 16.25) = 0.12, *p* =.809, η_p_^2^ =.010; direction × distance: *F*(8, 96) = 0.54, *p* =.822, η_p_^2^ =.043; direction × attention × distance: *F*(8, 96) = 1.41, *p* =.200, η_p_^2^ =.105. Further analysis of the interaction revealed that the effect of distance was significant for both attention conditions, near: *F*(4, 48) = 22.56, *p* <.001, η_p_^2^ =.653; far: *F*(4, 48) = 32.50, *p* <.001, η_p_^2^ =.730. Accordingly, multiple comparisons across distance conditions were conducted (Table 1). The Δ *d′* value was significantly larger at the attended distances in both attention conditions. Furthermore, simple main effects analyses of attention at each tested distance revealed significant effects at 32 cm, *F*(1, 12) = 118.41, *p* <.001, η_p_^2^ =.908; 128 cm, *F*(1, 12) = 5.05, *p* =.044, η_p_^2^ =.296; and 160 cm, *F*(1, 12) = 248.61, *p* <.001, η_p_^2^ =.954, indicating that sensitivity (Δ *d′*) differed significantly between the near and far attention conditions at these distances. No significant effect of attention was observed at 64 cm, *F*(1, 12) = 0.19, *p* =.673, η_p_^2^ =.015, or 96 cm, *F*(1, 12) = 0.01, *p* =.905, η_p_^2^ =.001. These findings indicate that Δ *d′* was highest at the attended distance and declined with increasing distance from that location.

**Table 1:** Results of multiple comparisons of Δ*d′* across distance pairs and attention conditions

Comparison	Near		Far	
	t value	Adj. *p* value	t value	Adj. *p* value
32 vs. 64	**9.93**	**< 0.001**	0.81	1.000
32 vs. 96	**6.24**	**< 0.001**	0.53	1.000
32 vs. 128	**9.90**	**< 0.001**	**4.07**	**< 0.001**
32 vs. 160	**8.88**	**0.003**	**7.88**	**< 0.001**
64 vs. 96	0.40	1.000	0.03	1.000
64 vs. 128	0.32	1.000	2.54	0.130
64 vs. 160	1.12	1.000	**9.41**	**< 0.001**
96 vs. 128	0.14	1.000	1.95	0.299
96 vs. 160	0.68	1.000	**9.07**	**< 0.001**
128 vs. 160	0.83	1.000	6.94	0.001

Boldface values indicate statistically significant comparisons (adjusted *p* <.05)

Exploratory one-sample Wilcoxon signed-rank tests against zero revealed significant deviations in several conditions (see Table 1). In the front space, significant deviations were observed at 32 cm in the near-attention condition (*V* = 91.00, *p* <.001) and at 160 cm in the far-attention condition (*V* = 90.00, *p* <.001). In the lateral space, significant deviations were found at 32 and 160 cm in the near-attention condition (32 cm: *V* = 90.00, *p* =.002; 160 cm: *V* = 14.00, *p* =.030), and at 32, 64, 96, and 160 cm in the far-attention condition (32 cm: *V* = 1.50, *p* =.004; 64 cm: *V* = 8.50, *p* =.018; 96 cm: *V* = 0.00, *p* =.036; 160 cm: *V* = 85.00, *p* =.003). In the rear space, significant deviations were observed at 32 and 64 cm in the near-attention condition (32 cm: *V* = 1.50, *p* =.004; 64 cm: *V* = 8.50, *p* =.018), and at 160 cm in the far-attention condition (*V* = 80.00, *p* =.013). These deviations included both positive and negative values relative to zero, indicating attentional enhancement and inhibition, respectively.

#### Reaction time

**Fig. 3:**
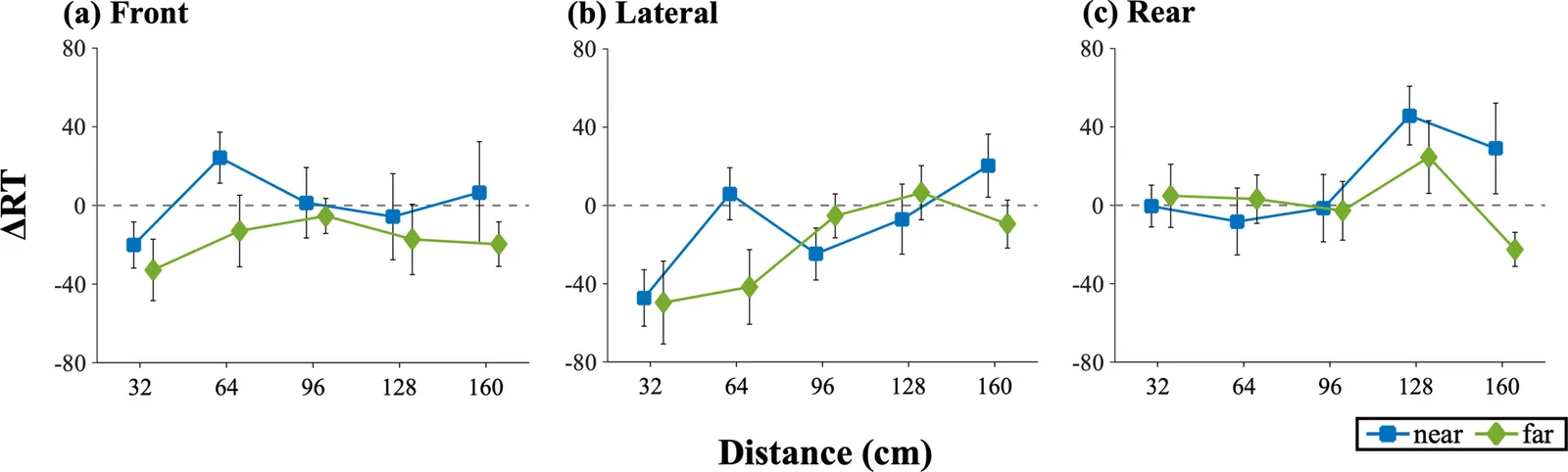
Difference in reaction time (ΔRT, ms) between the focus and no-focus conditions as a function of target distance. **(a)** Front condition, **(b)** lateral condition, and **(c)** rear condition. Error bars represent the standard error of the mean. For better readability, data points are slightly offset along the horizontal axis. (Color figure online)

Figure 3 shows the relative RT (ΔRT) as a function of tested distance for each condition. The ANOVA revealed a significant main effect of distance, *F*(4, 48) = 2.60, *p* =.047, η_p_^2^ =.178, as well as significant interactions between attention and distance, *F*(4, 48) = 2.73, *p* =.040, η_p_^2^ =.185, and among direction, attention, and distance, *F*(8, 96) = 2.27, *p* =.029, η_p_^2^ =.159. No other main effects or interactions reached significance, direction: *F*(2, 24) = 2.12, *p* =.142, η_p_^2^ =.150; attention: *F*(1, 12) = 2.62, *p* =.132, η_p_^2^ =.179; direction × attention: *F*(2, 24) = 0.30, *p* =.746, η_p_^2^ =.024; direction × distance: *F*(8, 96) = 1.31, *p* =.246, η_p_^2^ =.099.

Further analysis of the three-way interaction among direction × attention × distance and separate two-way ANOVAs (attention × distance) was conducted for each direction condition (i.e., front, lateral, and rear).

No significant main effects or interactions were found for the front condition, attention: *F*(1, 12) = 3.11, *p* =.103, η_p_^2^ =.206; distance: *F*(4, 48) = 0.79, *p* =.537, η_p_^2^ =.062; attention × distance: *F*(4, 48) = 0.59, *p* =.669, η_p_^2^ =.047.

For the lateral condition, there was a significant main effect of distance, *F*(4, 48) = 2.93, *p* =.030, η_p_^2^ =.197, and a significant interaction between attention and distance, *F*(4, 48) = 5.21, *p* =.001, η_p_^2^ =.303. Further analysis of the interaction revealed that the effect of distance was significant in both attention conditions, near: *F*(4, 48) = 3.90, *p* =.008, η_p_^2^ =.245; far: *F*(4, 48) = 2.97, *p* =.029, η_p_^2^ =.198; however, multiple comparisons did not reveal any statistically significant pairwise differences (*p* values >.064). Simple main effect analyses of attention at each tested distance revealed significant effects at 64 cm, *F*(1, 12) = 6.38, *p* =.027, η_p_^2^ =.347, and 160 cm, *F*(1, 12) = 5.93, *p* =.032, η_p_^2^ =.331, but not at the other distances, 32 cm: *F*(1, 12) = 0.04, *p* =.848, η_p_^2^ =.003; 96 cm: *F*(1, 12) = 0.86, *p* =.371, η_p_^2^ =.067; 128 cm: *F*(1, 12) = 0.98, *p* =.342, η_p_^2^ =.076. These results indicate that ΔRT was significantly shorter in the far-attention condition than in the near-attention condition at 64 and 160 cm.

A significant interaction between attention and distance was also found for the rear direction, *F*(4, 48) = 2.78, *p* =.037, η_p_^2^ =.188, whereas the main effects were not significant, attention: *F*(1, 12) = 1.05, *p* =.327, η_p_^2^ =.080; distance: *F*(4, 48) = 1.75, *p* =.154, η_p_^2^ =.128. Follow-up analyses of the interaction indicated that the effect of distance was not significant in either attention condition, near: *F*(2.54, 30.46) = 2.35, *p* =.101, η_p_^2^ =.164; far: *F*(4, 48) = 1.65, *p* =.176, η_p_^2^ =.121. Simple main effects analyses of attention at each tested distance revealed a significant effect at 160 cm (*F*(1, 12) = 6.63, *p* =.024, η_p_^2^ =.356), but not at the other distances, 32 cm: *F*(1, 12) = 0.16, *p* =.700, η_p_^2^ =.013; 64 cm: *F*(1, 12) = 0.33, *p* =.578, η_p_^2^ =.027; 96 cm: *F*(1, 12) = 0.01, *p* =.941, η_p_^2^ =.001; 128 cm: *F*(1, 12) = 1.63, *p* =.226, η_p_^2^ =.119. These results indicate that ΔRT was significantly shorter in the far-attention condition than in the near-attention condition at 160 cm.

Exploratory one-sample Wilcoxon signed-rank tests against zero also revealed significant deviations in several RT conditions. Significant deviations were observed in the front space at 32 cm in the far-attention condition (*V* = 17.00, *p* =.048); in the lateral space at 32 cm in both the near-attention (*V* = 8.00, *p* =.006) and far-attention conditions (*V* = 17.00, *p* =.048); and in the rear space at 128 cm in the near-attention condition (*V* = 82.00, *p* =.008) and at 160 cm in the far-attention condition (*V* = 15.00, *p* =.033). These deviations included both positive and negative values relative to zero, indicating attentional inhibition and enhancement, respectively.

### Auditory distance perception

**Fig. 4:**
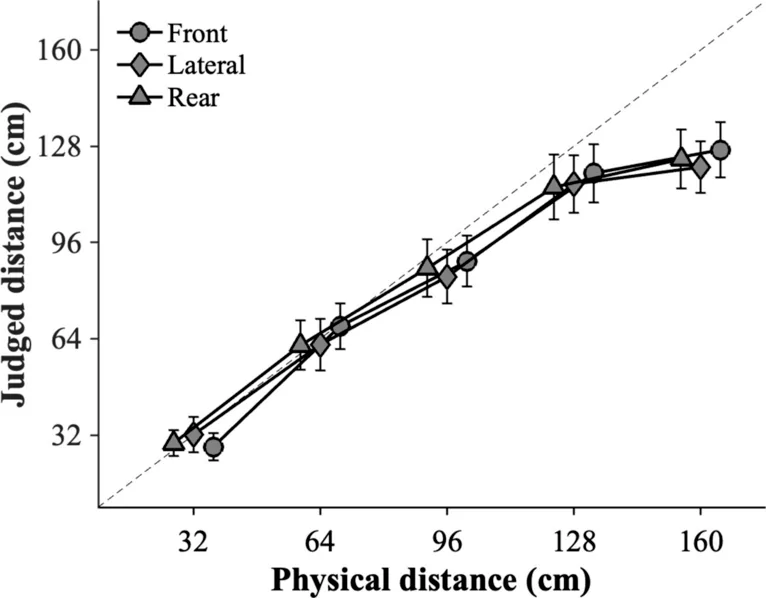
Average judged distance as a function of physical sound source distance for each direction. Error bars denote the standard error of the mean. The dotted line represents veridical judgments. For better readability, data points are slightly offset along the horizontal axis

Figure 4 shows the mean judged distance of the auditory stimuli as a function of the physical sound source distance. A two-way repeated-measures ANOVA revealed a significant main effect of distance, *F*(1.93, 23.2) = 172.75, *p* <.001, η_p_^2^ =.935, but no effect of direction, *F*(2, 24) = 0.48, *p* =.626, η_p_^2^ =.038, or interaction, *F*(4.02, 48.23) = 0.73, *p* =.579, η_p_^2^ =.057. Multiple comparisons of the effect of distance revealed significant differences between all combinations (*p *<.001), suggesting that the listeners could discriminate between the distance conditions. The absence of directional effects suggests that auditory distance perception did not depend on direction. Additionally, across all directions, judged distances tended to be underestimated relative to the physical distances at farther tested distances. This underestimation is consistent with previous findings (for reviews, Kolarik et al., 2016, 2025; Zahorik et al., 2005), which show that auditory distance perception becomes increasingly compressed as physical distance increases.

## Discussion

The present study aimed to investigate whether auditory attention in the depth dimension differs across directions, using sensitivity (*d′*) and RT as indices. Consistent with previous studies, directing attention to a particular distance enhanced sensitivity (*d′*) for target sounds at that distance, with the magnitude of the effect decreasing as targets were presented farther from the attended distance. Notably, the effect of attention did not differ significantly across directions. However, the results for RT were less consistent: attentional benefits were evident only in the far-attention condition and primarily in the lateral and rear spaces.

Recent studies have demonstrated that auditory selective attention enhances listening performance, such as the RT, and detection performance for the target sound, when directed to a specific distance in complex listening situations (Monasterolo, 2019; Sakamoto et al., 2019; Teraoka et al., 2025). Teraoka et al. (2025) investigated the influence of auditory attention on target detection in the depth dimension in the front space using the same experimental paradigm as the present study. Their results showed that directing attention to a specific distance significantly increased the sensitivity (*d′*) at that distance compared to other distances. The present study replicated this effect in the front condition. Additionally, sensitivity to the target sound decreased as the distance from the attended position increased. This pattern indicates that attentional effects are spatially distributed, with the strongest enhancement at the attended distance, followed by a sharp reduction at the nearest nonattended distance and a relatively shallow or stable profile across more distant locations.

One of the most important contributions of the present study lies in its extension to include lateral and rear spaces. Sensitivity (*d′*) was highest at the attended distance and decreased as the distance from that location increased; however, this pattern did not differ significantly across directions. This finding suggests no clear directional dependency in the effects of auditory attention in the depth dimension. Instead, the results are more consistent with the hypothesis that auditory attention in depth is similarly organized across directions around the body than with the alternative hypothesis that it varies systematically with direction.

Previous studies have demonstrated that auditory spatial attention enhances listening performance. Most of these studies have been conducted in the horizontal plane, with a predominant focus on the frontal space (Arbogast & Kidd, 2000; Teder-Sälejärvi & Hillyard, 1998; Teraoka et al., 2021). Only a few studies have directly examined whether attention effects vary across other horizontal directions, such as lateral or rear spaces. Teder-Sälejärvi et al. (1999) compared the spatial profile of auditory attention between the front and lateral spaces and observed a shallower attentional gradient in the lateral direction, suggesting a more diffuse distribution of attention. Similarly, Bodnár et al. (2025) compared the front and rear spaces and reported a steeper attentional gradient in the front than in the rear space. However, the present study did not provide clear evidence for such directional dependency in the effects of auditory attention in the depth dimension.

Although no significant interaction between direction and attention was found, this result should be interpreted with caution. The absence of a statistical interaction suggests that the gradient of auditory attention in the depth dimension was comparable across spatial directions within the present experimental design. However, considering previous studies on auditory selective attention in the horizontal plane, it is reasonable to expect that attentional effects in depth may differ across the front, lateral, and rear spaces. One possible explanation for the absence of direction-dependent differences is that the 32-cm spacing between adjacent loudspeakers may have been too coarse to detect subtle variations in the attentional profile across depth. If the attentional focus in depth was relatively broad, attentional enhancement may have spread across adjacent distance locations. In this case, subtle directional differences in the width or slope of the attentional profile may have been obscured by the spatial resolution of the experimental setup.

Another important consideration is the role of peripersonal space. Peripersonal space (PPS) refers to the immediate space surrounding the body and is thought to support interactions with nearby objects (for a review, Serino, 2019). Previous studies suggest that PPS extends to approximately 70 cm from the body surface (e.g., Serino et al., 2015), and that attentional processing may be facilitated within this space relative to more distant regions (Losier & Klein, 2004; Reed & Park, 2021). Thus, the distance range used in the present study (32–160 cm) likely encompassed both peripersonal and extrapersonal space. If so, the observed differences between near and far positions may reflect not only the spatial profile of attention in depth but also differences in the processing of near and far space. Notably, because the present analysis relied on difference scores relative to the no-focus condition, some baseline differences between the conditions may have been partially controlled. However, if peripersonal and extrapersonal spaces differ not only in baseline processing but also in how attentional effects are expressed, such influences may still have contributed to the observed pattern. Furthermore, because previous studies have reported directional asymmetries in PPS-related processing (Amiel et al., 2025; Hobeika et al., 2018; Teraoka et al., 2024), these factors may have masked subtle directional variations in the spatial profile observed here. Future studies should therefore examine auditory attention in depth while directly assessing PPS representations.

Additionally, the exploratory analyses revealed that some conditions, particularly those at distances away from the attended position, showed negative deviations relative to the no-focus condition. This pattern suggests that the observed effects were not limited to enhanced sensitivity at the attended distance but may also involve reduced sensitivity at certain nonattended distances. This interpretation is broadly consistent with accounts proposing that auditory spatial attention involves both facilitation at attended locations and inhibition of irrelevant information, which may occur concurrently (Bodnár et al., 2025; see also Klein, 2000; Posner & Cohen, 1984). However, because these negative deviations were limited, condition-specific, and not consistently observed across directions or distances, they should be interpreted cautiously and do not constitute strong evidence of inhibition. At the same time, their nonuniform distribution across directions may suggest subtle directional anisotropy, although the present data are insufficient to support a firm conclusion.

The RT results partially diverged from those for sensitivity. In the front condition, no significant RT effects were observed at any tested distance. This absence of RT effects in the front space is consistent with a previous study (Teraoka et al., 2025), which employed the same paradigm to investigate auditory attention in the depth dimension. This pattern suggests that the effects of auditory attention may be less pronounced in the front space, at least in terms of response speed.

Significant RT facilitation was observed in the lateral and rear spaces at specific distances (64 and 160 cm in the lateral space, and 160 cm in the rear space), whereas no significant effects were observed in the near-attention condition for any direction. Although these effects were limited, they suggest that auditory attention may yield greater benefits in non-frontal spaces, where reliance on auditory information is inherently greater due to the lack of visual access.

The absence of RT facilitation in the near condition across all directions may be attributable to a floor effect. Target sounds presented at near distances were detected with high accuracy and short RTs, thereby minimizing the potential for additional response speeding through attentional enhancement. Furthermore, RT is a composite measure that reflects both perceptual/decisional processing time and motor execution time (e.g., Prinzmetal et al., 2005). Variability in these components may introduce noise that masks subtle attentional effects at the perceptual stage. In contrast, *d′* more directly reflects perceptual sensitivity, allowing it to capture subtle changes in processing efficiency that may not be large enough to significantly affect overall reaction time.

The RT advantage observed in the far-attention condition for the lateral and rear spaces likely reflects an ecological asymmetry in spatial monitoring. The frontal space is constantly monitored by vision and audition, whereas the lateral and rear spaces lie outside the visual field, and spatial judgements rely mainly on auditory cues. Consequently, attention may be more readily directed to auditory events in these nonfrontal spaces. Consistent with this idea, several studies have reported that sounds orienting from behind or outside the visual field attract stronger attention and are perceived as more behaviorally significant than frontal sounds (Asutay & Västfjäll, 2015; Bodnár et al., 2025; Olszanowski et al., 2023; Teraoka et al., 2024).

These results suggest that auditory attention in the depth dimension may exhibit spatial anisotropy at the behavioral level, with relatively greater facilitation in lateral and rear spaces. However, given that these effects were modest and observed only at specific distances, further investigation using complementary behavioral and neural measures will be necessary to confirm the robustness and clarify the underlying mechanisms of this spatial pattern.

The present study has several limitations. First, it was conducted in a reverberant room characterized by reflective surfaces such as walls and floor carpeting. While this setting enhances ecological validity by approximating real-world listening conditions, it also means that multiple auditory distance cues were available. Accordingly, the present results should be interpreted as reflecting attentional effects under relatively natural listening conditions, rather than the minimal effects expected from direct sound alone in a more acoustically controlled environment. Although the use of Δ*d′* and ΔRT likely reduced acoustic influences shared across conditions, the present design does not dissociate the specific depth cues contributing to the observed attentional effects. Future studies conducted in acoustically controlled environments (e.g., anechoic rooms) are therefore needed to evaluate the robustness and generalizability of these findings.

Second, the probability-based manipulation used in the present study does not allow for a clear isolation of endogenous attention. In the focus condition, listeners were not explicitly instructed to attend to a particular distance; instead, attention was biased through a probability manipulation in which sounds were presented more frequently at one distance than at others. This approach was adopted based on previous auditory attention studies using similar designs (e.g., Arbogast & Kidd, 2000; Teraoka et al., 2021, 2025). More broadly, prior research suggests that probabilistic information can guide endogenous spatial attention (e.g., Posner, 1980; Summerfield & Egner, 2009; Zuanazzi & Noppeney, 2019). However, recent accounts emphasize that attentional orienting is shaped not only by endogenous and exogenous factors but also by selection history, that is, prior experience with frequently occurring events (for reviews, Anderson et al., 2021; Awh et al., 2012). In the present paradigm, stimuli were presented from a particular distance on 80% of trials, which may have led listeners to form expectations and allocate attention accordingly. Therefore, the results cannot be interpreted as reflecting purely endogenous attention. Future studies should employ designs that more clearly dissociate endogenous and history-driven components of attentional orienting.

## Conclusions

The present study provides clear evidence that auditory attention directed along the depth dimension enhances target detection performance at the attended distance, irrespective of the spatial direction from which sounds are presented. The magnitude of the attentional benefit in sensitivity was comparable across front, lateral, and rear spaces. While the RT data showed some effects in the lateral and rear directions, these effects were not consistent across conditions. Overall, these results indicate that the auditory system flexibly allocates spatial attention in the depth dimension without a strong directional bias.

## Supplementary Information

Below is the link to the electronic supplementary material.

Supplementary file1 (DOCX 1575 KB)

## Data Availability

The analysis is available from the corresponding author upon reasonable request.

## Footnotes

1 This procedure was adopted to encourage endogenous auditory attention toward a specific position, following previous studies and literature linking probabilistic information to endogenous spatial attention (e.g., Posner, 1980; Summerfield & Egner, 2009; Teraoka et al., 2025; Zuanazzi & Noppeney, 2019); however, such probability-based manipulations may reflect multiple influences, including goal-directed attentional orienting and selection history (for reviews, see Anderson et al., 2021; Awh et al., 2012). The present design was not intended to dissociate these components (see the Discussion section for additional details).
