# Supplementary material for: Auditory selective attention in depth: Investigating directional dependency across front, lateral, and rear spaces
Source: Atten Percept Psychophys. 2026 Jun 23;88(6):152. doi: 10.3758/s13414-026-03292-x (PMC13291049; doi:10.3758/s13414-026-03292-x)
Supplement: Supplementary file 1 — Supplementary file1 (DOCX 1575 KB) [file 13414_2026_3292_MOESM1_ESM.docx]

**Supplementary analysis**

In the main text, we focused on the attentional modulation of listening performance by subtracting baseline (no-focus) performance. This approach was intended to isolate attentional effects from physical distance-dependent factors that inherently influence auditory performance. To provide a more comprehensive account of listener behavior, the present supplementary analyses examine the absolute values of sensitivity (*d’*) and reaction time (RT) across all conditions. Furthermore, we computed response bias (criterion *c*) to determine whether the observed effects reflect improved listening performance or changes in response strategy.

**Methods**

**Data analysis**

Statistical analyses were performed using R (version: 4.4.2) with the *anovakun* package (Iseki, 2024). Sensitivity (d’) and response bias (criterion *c*) were computed from hit (*H*) and false alarm (*FA*) rates based on Signal Detection Theory, using the following formulae:

$$d^{'}=z\left( H \right)-z(FA)$$

$$c=-0.5\left[ z\left( H \right)+z\left( FA \right) \right]$$

where *z* denotes the z-score transformation. A *c* value of 0 indicates an unbiased response criterion, whereas positive and negative values indicate conservative and liberal bias, respectively [Macmillan & Creelman, 2005]. Additionally, reaction time (RT) was analyzed to evaluate the speed and efficiency of target detection.

For *d’*, RT, and *c*, a three-way repeated-measures analysis of variance (ANOVA) was conducted on the mean data, with attention (3 levels: no-focus, near, and far), direction (3 levels: front, lateral, and rear), and distance (5 levels: 32, 64, 96, 128, and 160 cm) as within-subject factors. Multiple comparisons were performed using Holm’s correction, a sequentially rejective Bonferroni (SRB) procedure, with the family-wise significance level set at α = .05.

**Results and Discussion**

***Sensitivity (d’)***

Figure S1 shows sensitivity (*d’*) as a function of tested distance for each condition. The ANOVA revealed a significant main effect of distance (*F* (4, 48) = 15.65, *p* < .001, η_p_^2^ = .566) and a significant interaction between attention and distance (*F* (8, 96) = 46.98, *p* < .001, η_p_^2^ = .797), but no other significant main effects or interactions (direction: *F* (2, 24) = 1.78, *p* = .190, η_p_^2^ = .129; attention: *F* (2, 24) = 0.38, *p* = .688, η_p_^2^ = .031; direction × attention: *F*(2.09, 25.1) = 0.45, *p* = .655, η_p_^2^ = .036; direction × distance: *F*(8, 96) = 0.63, *p* = .752, η_p_^2^ = .050; direction × attention × distance: *F*(16, 192) = 0.95, *p* = .521, η_p_^2^ = .073).

Follow-up analyses of the interaction revealed a significant main effect of attention at 32 cm and 160 cm (32 cm: *F*(2, 24) = 73.43, *p* < .001, η_p_^2^ = .860; 160 cm: *F*(2, 24) = 87.84, *p* < .001, η_p_^2^ = .880), but not at the other distances (64 cm: *F*(2, 24) = 3.15, *p* = .061, η_p_^2^ = .208, 96 cm: *F*(2, 24) = 2.57, *p* = .097, η_p_^2^ = .176, 128 cm: *F*(2, 24) = 2.78, *p* = .082, η_p_^2^ = .188). Multiple comparisons revealed significant differences among all attention conditions at both 32 cm and 160 cm. At 32 cm, *d’* followed the pattern near > no-focus > far (*p*s < .006), whereas at 160 cm the pattern was reversed (far > no-focus > near; *p*s < .002).

Further analyses revealed a significant main effect of distance within each attention condition (no-focus: *F*(4, 48) = 2.97, *p* = .029, η_p_^2^ = .198; near: *F*(4, 48) = 57.81, *p* < .001, η_p_^2^ = .828; far: *F*(4, 48) = 35.71, *p* < .001, η_p_^2^ = .749). Table S1 summarizes the results of post hoc multiple comparisons across distances within each attention condition. These comparisons showed that *d’* was significantly higher at the attended distance under focused attention conditions, whereas no significant differences across distances were observed in the no-focus condition.

These results indicate that sensitivity (*d’*) was highest at the attended distance. Specifically, *d’* differed significantly from the no-focus condition only at the focus distances, indicating enhanced sensitivity at attended relative to unattended distances. This pattern suggests that listeners successfully allocated attentional resources to the target distance. Because this effect was observed at both 32 cm and 160 cm, selective attention in depth appears to modulate perceptual sensitivity robustly, regardless of whether the attended location is near or far.

**Figure S1**

*Sensitivity (d’) as a Function of Target Distance*

*Note.* (a) Front condition, (b) lateral condition, and (c) rear condition. Error bars represent the standard error of the mean. For clarity, data points are slightly offset along the horizontal axis.

**Table S1**

*Results of Multiple Comparisons of d’ for Each Pair of Distances and Attention Condition*

**

*Note.* Bold values indicate statistically significant comparisons (adjusted *p* < .05).

***Reaction Time***

Figure S2 shows the reaction time (RT) as a function of the tested distance for each condition. The ANOVA revealed a significant main effect of distance (*F*(2.34, 28.12) = 41.40, *p* < .001, η_p_^2^ = .775) and significant interactions for attention × distance (*F*(8, 96) = 2.68, *p* = .011, η_p_^2^ = .182) and direction × attention × distance (*F*(16, 192) = 1.76, *p* = .039, η_p_^2^ = .128). No other main effects or interactions were significant (direction: *F*(2, 24) = 0.29, *p* = .747, η_p_^2^ = .024; attention: *F*(1.39, 16.68) = 2.36, *p* = .137, η_p_^2^ = .164; direction × attention: *F*(4, 48) = 1.20, *p* = .323, η_p_^2^ = .091; direction × distance: *F*(8, 96) = 0.57, *p* = .797, η_p_^2^ = .046).

To further examine the three-way interaction (direction × attention × distance), separate two-way ANOVAs (attention × distance) were conducted for each direction (front, lateral, and rear).

For the front condition, a significant main effect of distance was observed (*F*(4, 48) = 19.47, *p* < .001, η_p_^2^ = .619), whereas neither the main effect of attention (*F*(2, 24) = 2.28, *p* = .124, η_p_^2^ = .160) nor the interaction (attention × distance: *F*(8, 96) = 0.69, *p* = .701, η_p_^2^ = .054) was significant. Pairwise comparisons for distance (Table S2) showed that RTs at 32 cm were significantly shorter than those at 64, 96, 128, and 160 cm (*p*s < .002), and that RTs at 64 cm were significantly shorter than those at 128 and 160 cm (*p*s < .031). No other pairwise differences were significant.

For the lateral and rear conditions, there were significant main effects of distance (lateral: *F*(4, 48) = 23.68, *p* < .001, η_p_^2^ = .664; rear: *F*(2.35, 28.24) = 25.14, *p* < .001, η_p_^2^ = .677) and significant attention × distance interactions (lateral: *F*(8, 96) = 3.94, *p* < .001, η_p_^2^ = .247; rear: *F*(8, 96) = 2.32, *p* = .025, η_p_^2^ = .162), but no main effect of attention (lateral: *F*(2, 24) = 1.92, *p* = .168, η_p_^2^ = .138; rear: *F*(2, 24) = 0.98, *p* = .389, η_p_^2^ = .076). The results of post hoc comparisons across distances within each attention condition are summarized in Table S3. These comparisons indicate that RT was primarily influenced by distance. Although some distance-dependent variations across attention conditions were observed in the lateral and rear directions, these effects were small and did not reveal consistent facilitation at the attended distance.

Overall, these results indicate that, unlike sensitivity (*d’*), RT increased as a function of target distance regardless of attention condition. This suggests that RT was primarily driven by the physical distance of the stimuli rather than attentional focus. The increase in RT for more distant targets likely reflects reduced signal strength (i.e., sound attenuation). Furthermore, the absence of clear attentional facilitation in RT may be explained by factors such as a floor effect at near distances or variability in motor and decisional processes. These factors may have masked subtle attentional enhancements that were captured by the sensitivity measure (*d’*).

**Figure S2**

*Reaction Time (RT) as a Function of Target Distance*

*Note.* (a) Front condition, (b) lateral condition, and (c) rear condition. Error bars represent the standard error of the mean. For clarity, data points are slightly offset along the horizontal axis.

**Table S2**

*Results of Multiple Comparisons of RT Between Distances for the Front Condition*

*Note.* Bold values indicate statistically significant comparisons (adjusted *p* < .05).

**Table S3**

*Results of Multiple Comparisons of RT across Distance Pairs and Each Attention Condition in the Lateral and Rear Directions.*

**

*Note.* Bold values indicate statistically significant comparisons (adjusted *p* < .05).

***Response bias (c)***

Figure S3 shows response bias (*c*) as a function of tested distance for each condition. The ANOVA revealed significant main effects of direction (*F*(2, 24) = 3.77, *p* = .038, η_p_^2^ = .239), attention (*F*(2, 24) = 9.07, *p* = .001, η_p_^2^ = .431), and distance (*F*(4, 48) = 18.03, *p* < .001, η_p_^2^ = .601), as well as significant interaction between attention and distance (*F*(8, 96) =3.94, *p* = .001, η_p_^2^ = .247). No other interactions were significant (direction × attention: *F*(4, 48) = 0.93, *p* = .457, η_p_^2^ = .072; direction × distance: *F*(8, 96) = 1.53, *p* = .153, η_p_^2^ = .114; direction × attention × distance: *F*(16, 192) = 0.89, *p* = .580, η_p_^2^ = .069). Multiple comparisons for the effect of direction did not reveal any significant pairwise differences (*p*s > .117).

Follow-up analyses of the attention × distance interaction showed a significant effect of attention at 32 cm (*F*(2, 24) = 16.17, *p* < .001, η_p_^2^ = .574) and 64 cm (*F*(2, 24) = 7.77, *p* = .003, η_p_^2^ = .393), but not at the other distances (96 cm: *F*(2, 24) = 2.04, *p* = .152, η_p_^2^ = .145; 128 cm: *F*(2, 24) = 1.16, *p* = .331, η_p_^2^ = .088; 160 cm: *F*(2, 24) = 1.98, *p* = .160, η_p_^2^ = .142). At 32 cm, *c* was significantly lower in the far-attention condition than in the other conditions, whereas the no-focus and near-attention conditions did not differ significantly. At 64 cm, *c* was significantly higher in the near-attention condition than in the other conditions, whereas the no-focus and far-attention conditions did not differ significantly.

Further analyses revealed a significant main effect of distance within each attention condition (no-focus: *F*(4, 48) = 6.90, *p* < .001, η_p_^2^ = .365; near: *F*(4, 48) = 5.59, *p* = .001, η_p_^2^ = .318; far: *F*(4, 48) = 17.82, *p* < .001, η_p_^2^ = .598). Multiple comparisons showed that, in the no-focus condition, *c* at 32 cm was significantly lower than at 160 cm. In the near-attention condition, *c* at 32 cm was significantly lower than that at 64 cm. In the far-attention condition, *c* at 32 cm was significantly lower than that at all other tested distances.

These results suggest that response bias (*c*) varied systematically as a function of target distance, generally increasing for more distant targets. This pattern indicates that listeners adopted a more conservative decision criterion for targets presented at greater distances. One possible explanation is that increased distance introduced greater perceptual uncertainty, thereby shifting the decision criterion in a more conservative direction and reducing the likelihood of reporting a target. Although the pattern of *c* varied somewhat across spatial directions, these differences were limited and did not indicate robust directional modulation. Overall, these findings suggest that response bias was influenced more strongly by target distance than by attentional focus.

**Figure S3**

*Response Bias (c) as a Function of Target Distance*

*Note.* (a) Front condition, (b) lateral condition, and (c) rear condition. Error bars represent the standard error of the mean. For clarity, data points are slightly offset along the horizontal axis.
